# Calcium signaling in oocyte quality and functionality and its application

**DOI:** 10.3389/fendo.2024.1411000

**Published:** 2024-08-16

**Authors:** Chen Chen, Zefan Huang, Shijue Dong, Mengqian Ding, Jinran Li, Miaomiao Wang, Xuhui Zeng, Xiaoning Zhang, Xiaoli Sun

**Affiliations:** ^1^ Institute of Reproductive Medicine, Medical School, Nantong University, Nantong, China; ^2^ Center for Reproductive Medicine, Affiliated Hospital of Nantong University, Nantong University, Nantong, China

**Keywords:** calcium, oocyte maturation, oocyte activation, fertilization, Ca^2+^ oscillations, female fertility

## Abstract

Calcium (Ca^2+^) is a second messenger for many signal pathways, and changes in intracellular Ca^2+^ concentration ([Ca^2+^]i) are an important signaling mechanism in the oocyte maturation, activation, fertilization, function regulation of granulosa and cumulus cells and offspring development. Ca^2+^ oscillations occur during oocyte maturation and fertilization, which are maintained by Ca^2+^ stores and extracellular Ca^2+^ ([Ca^2+^]e). Abnormalities in Ca^2+^ signaling can affect the release of the first polar body, the first meiotic division, and chromosome and spindle morphology. Well-studied aspects of Ca^2+^ signaling in the oocyte are oocyte activation and fertilization. Oocyte activation, driven by sperm-specific phospholipase PLCζ, is initiated by concerted intracellular patterns of Ca^2+^ release, termed Ca^2+^ oscillations. Ca^2+^ oscillations persist for a long time during fertilization and are coordinately engaged by a variety of Ca^2+^ channels, pumps, regulatory proteins and their partners. Calcium signaling also regulates granulosa and cumulus cells’ function, which further affects oocyte maturation and fertilization outcome. Clinically, there are several physical and chemical options for treating fertilization failure through oocyte activation. Additionally, various exogenous compounds or drugs can cause ovarian dysfunction and female infertility by inducing abnormal Ca^2+^ signaling or Ca^2+^ dyshomeostasis in oocytes and granulosa cells. Therefore, the reproductive health risks caused by adverse stresses should arouse our attention. This review will systematically summarize the latest research progress on the aforementioned aspects and propose further research directions on calcium signaling in female reproduction.

## 1 Introduction

As one of the most important second messengers vital for cellular signaling and homeostasis in cells (1), Ca^2+^ is involved in regulating almost all biological functions of the body, such as fertilization, proliferation, development, learning and memory, contraction and secretion (2). To perform these functions, free Ca^2+^ is present in different concentrations in the organelle, cytoplasm, nucleus and extracellular microenvironment. In most cell types, the concentrations of Ca^2+^ are about 100 nM in the resting state, while the extracellular concentrations of Ca^2+^ are typically 1-2 mM (1) and endoplasmic reticulum (ER) concentrations of Ca^2+^ are approximately 0.5-1 mM (3). Unlike other second messengers, Ca^2+^ cannot be metabolized and is essential for life, so cells precisely regulate [Ca^2+^]i through calcium ion channels, abundant calcium buffering proteins and specialized calcium extrusion proteins such as calcium pumps (1).

Ca^2+^ signaling is fundamental to oocyte maturation and activation (4). In all studied animals, oocytes are arrested in the meiotic prophase I while they undergo growth and differentiation. In this stage, the oocyte nucleus is large and is termed the germinal vesicle (GV), hence, oocytes at this stage are also called GV oocytes. The GV oocytes remodel the Ca^2+^ signaling machinery (5) and store macromolecular components required for fertilization of oocytes and early embryonic development (6). Following hormonal stimulation, the oocyte emerges from this prolonged meiotic arrest and undergoes a complex differentiation pathway involving both meiosis and extensive cytoplasmic rearrangement. This prepares the oocyte for its transition from egg to embryo after fertilization (7). Ca^2+^ signaling initiating development has been extensively studied in many mammalian and non-mammalian eggs (8–10). Ca^2+^ channels or Ca^2+^ transporters in the cell membrane regulate multiple aspects of meiotic maturation and fertilization of mammalian oocytes by controlling the spatiotemporal distribution of Ca^2+^, such as the resumption of meiotic maturation, polymerization, and depolymerization of microtubules, assembly and disassembly of spindle bodies, and withdrawal of meiosis after fertilization. The role of Ca^2+^ in egg maturation, fertilization and the application of Ca^2+^ signaling in assisted reproduction have been well described in several reviews (4, 11–13) over the past few years. Therefore, the aforementioned three sections have been succinctly summarized and updated in our paper. The following three parts will be discussed in detail, focusing on calcium homeostasis in granulosa cells (GC) and cumulus cells (CC), partners and pathways in oocyte Ca^2+^ signaling and adverse stress-induced calcium signaling related ovarian dysfunction.

## 2 Roles for Ca^2+^ in oocyte maturation

In mice (14) and humans (15), most GV-intact oocytes exhibit persistent Ca^2+^ oscillations, which may be related to the cell cycle and germinal vesicle breakdown (GVBD). In addition, the [Ca^2+^]i increase caused by the surge of follicle-stimulating hormone (FSH) (16) and other factors may also be involved in GVBD, recovery of oocyte meiosis (17, 18), and ovulation. Since calcium signaling is essential for oocyte maturation, we will divide this content into two parts, including (1) the function of Ca^2+^ during oocyte maturation (GV-MII) and (2) the source and regulation of Ca^2+^ during oocyte maturation.

### 2.1 Function of Ca^2+^ during oocyte maturation (GV-MII)

The development of oocytes from GV stage to metaphase I is closely related to increased [Ca^2+^]i. Previous studies have reported that follicle-stimulating hormone receptor (FSHR) may be present in oocytes (19). FSH binding to oocyte FSHRs was capable of mobilizing [Ca^2+^]i elevation that could be one of the initiators for the initiation of GVBD (16). In a recent study, it was reported that FSH could also induce an increase of [Ca^2+^]i in the GC (20). Similar to FSH, the Gonadotrophin-releasing hormone (GnRH) stimulated a transient increase in [Ca^2+^]i in rat GC (21). However, the significance of these increases in oocyte maturation requires further investigation. Different from FSH and GnRH, estrogen has adverse effects on spontaneous Ca^2+^ oscillations. It was reported that estrogen shortened the duration of spontaneous Ca^2+^ oscillations in a dose-dependent manner (1-1000 nM), and produced an irregular pattern of the oscillations (22). Whether spontaneous Ca^2+^ oscillations in the GV phase are necessary for oocytes to enter the GVBD phase remains controversial. Studies in mouse oocytes have shown that spontaneous Ca^2+^ oscillations in GV oocytes were absent when [Ca^2+^]i was chelated using 1,2-bis(o-aminophenoxy)ethane-N,N,N’,N’-tetraacetic acid tetra(acetoxymethyl) ester (BAPTA)-AM, but this did not affect the occurrence of GVBD. This result implies that spontaneous Ca^2+^ oscillations in GV oocytes and GVBD occurrence do not seem to be causally related in mice (14, 23). In contrast, in pig oocytes (24, 25), BAPTA-AM treatment inhibited GVBD, suggesting that elevated [Ca^2+^]i is a prerequisite for GVBD and meiotic progression. Therefore, the role of spontaneous Ca^2+^ oscillations in GVBD, even if redundant, cannot be ruled out. These differences in response may be due to the large variation in spontaneous maturation rates between species (26).

The contribution of Ca^2+^ involvement in GVBD is still controversial (23–25), but there is a consensus on the important role of Ca^2+^ in oocyte meiosis. It has been shown that incubation of GV oocytes in a matrix lacking external Ca^2+^ (23) or blocking Ca^2+^ channels with verapamil can inhibit first polar body formation (24, 27–29). Although the first polar bodies did not form after incubating GV oocytes in the absence of external Ca^2+^, oocytes were not prevented from progressing through meiosis I (23, 30). When high concentrations of Ca^2+^ chelators were used to bind [Ca^2+^]i, mouse oocyte meiosis was completely inhibited. This suggests that elevated [Ca^2+^]i promotes the recovery of oocyte meiosis (23, 30). The proper [Ca^2+^]i is also necessary for oocyte maturation. When the bovine MII-stage cumulus-oocyte complexes (COCs) were treated with BAPTA-AM, the intracellular [Ca^2+^]i was significantly lower. The decrease in cytoplasmic Ca^2+^ halted the nuclear maturation of oocytes, impaired cytoplasmic maturation, and diminished the developmental potential of somatic cell nuclear transfer embryos. These results indicate that Ca^2+^ is an important factor affecting the maturation of bovine oocytes (31).

### 2.2 Source and regulation of Ca^2+^ during oocyte maturation

#### 2.2.1 ER stores

ER stores are one of the most important sources of Ca^2+^ during oocyte maturation. ER are divided into smooth endoplasmic reticulum (SER) and granular endoplasmic reticulum (GER). SER’s role in calcium storage and release and GER’s role in protein synthesis are critical during oocyte maturation. In ER stores, inositol 1,4,5-trisphosphate receptors (IP3Rs) are the primary Ca^2+^ release channels. Most GV-intact oocytes isolated from mice’s antral follicles exhibit persistent and regular [Ca^2+^]i oscillations (14), which may be related to the cell cycle and GVBD. To investigate the source of Ca^2+^ in spontaneous Ca^2+^ oscillations, the researchers continuously injected inositol 1,4,5-trisphosphate (IP3) into mouse oocytes that spontaneously stopped Ca^2+^ oscillations. As a result, the oocytes produced new Ca^2+^ oscillations that were similar in amplitude and frequency to spontaneous Ca^2+^ oscillations. Moreover, spontaneous Ca^2+^ oscillations can be blocked by the injection of heparin, an IP3 receptor antagonist (32). These results suggest that IP3-induced Ca^2+^ release is the primary mechanism responsible for spontaneous Ca^2+^ oscillations during oocyte maturation (32).

Investigations have shown that Ca^2+^ release from eggs significantly increased when IP3 was artificially injected compared to GV oocytes (9, 33–35). During oocyte maturation, increased cellular sensitivity to IP3, redistribution of organelles such as the ER, and increased [Ca^2+^]i stores may contribute to variations in Ca^2+^ response. Increased sensitivity to IP3-dependent Ca^2+^ release during oocyte maturation is a hallmark of Ca^2+^ signaling differentiation in many species, including starfish (36), hamsters (37), mice (33), and Xenopus (35). There are three isoforms of IP3R, of which IP3R1 is closely associated with increased IP3 sensitivity. In mice, the protein level of IP3R1 approximately doubled from GV oocytes to MII oocytes (38). Inhibition of the increase in IP3R1 during oocyte maturation decreased the sensitivity of IP3 and shortened the duration of Ca^2+^ oscillations after fertilization (39). In addition, increased IP3 sensitivity may be associated with the phosphorylation of IP3R1 (9, 40, 41), which enhanced channel conductivity. Cdk1 may be essential for IP3R1 phosphorylation. Research has shown that the Cdk1 consensus sites, ser421 (S421) and threonine799 (T799), undergo phosphorylation in mouse oocytes at the MII stage (9). In somatic cells, phosphorylation of IP3R1 at these sites increased IP3 binding and IP3-gated Ca^2+^ release (42). In addition, in mouse eggs, expression of heterologous IP3R1 with a phosphorylation mutation corresponding to three M-phase motifs (which should be phosphorylated by M-phase kinase in MII eggs) resulted in greater Ca^2+^ oscillatory activity and sensitivity to IP3 (41). Besides M-phase kinases, studies in somatic cells have shown that IP3R isoforms can be phosphorylated by more wide-ranging kinases (43). The most commonly implicated kinases include protein kinase A (PKA), protein kinase C (PKC), and CaMKII, all of which have important physiological functions in oocytes and eggs. In mouse oocytes, IP3R1 phosphorylation by PKA, was shown during maturation, with maximal PKA phosphorylation occurring at the GV stage (9).

IP3Rs are also Ca^2+^ channel proteins that regulate the dynamic balance between the ER and mitochondrial Ca^2+^. A recent study found that inhibition of IP3R1 expression led to ER dysfunction, contributing to ER calcium release outwards into mitochondria and causing mitochondrial free calcium concentration overload and mitochondrial oxidative stress. Further analysis revealed that IP3R1 functioned by regulating the IP3R1-GRP75-VDAC1 complex between mitochondria and the ER (44). The loss of IR3R1 activity resulted in the failure of porcine oocyte maturation and CC expansion, as well as the obstruction of polar body excretion. These studies suggest that IP3Rs, especially IP3R1, play an important role in egg maturation. To further validate the role of IP3R1 in physiological processes, oocyte-specific knockout (KO) mice are needed.

It is also important to release Ca^2+^ from ER stores at the appropriate time. Premature Ca^2+^ release can lead to parthenogenetic activation before fertilization, making it crucial to prevent improper Ca^2+^ signaling to ensure oocyte MII arrest. Purinergic receptor P2Y2 (P2Y2R) is a G protein-coupled receptor that can induce the opening of IP3R on the ER, causing the release of Ca^2+^ from Ca^2+^ stores and leading to an increase in [Ca^2+^]i. It was found that the inhibition of P2Y2R during cryoprotectant exposure reduced premature [Ca^2+^]i release and significantly improved the developmental capacity of exposed bovine oocytes (45). Similar to P2Y2R, Regulators of G-protein signaling 2 (RGS2) was also found to inhibit premature Ca^2+^ release in MII-phase oocytes. *Rgs2^-/-^* female mice showed decreased litter size and premature zona pellucida transformation of eggs, suggesting that RGS2 is an important regulatory molecule that inhibits premature Ca^2+^ release from oocytes before fertilization (46). Thus, preventing the premature release of Ca^2+^ during oocyte maturation is essential for normal fertilization and early embryonic development. Taken together, these studies imply that ER Ca^2+^ stores are a very important source of Ca^2+^ increase and the proper Ca^2+^ release from ER stores is vital for the oocyte maturation.

#### 2.2.2 Extracellular fluid

Recent studies have found that [Ca^2+^]e is vital for spontaneous Ca^2+^ oscillations and the maturation of oocytes. It has been reported that spontaneous Ca^2+^ oscillations in oocytes are reversibly inhibited in the absence of [Ca^2+^]e (22). When the medium lacked Ca^2+^, there was a significant increase in intracytoplasmic Ca^2+^ in oocytes, leading to oocyte death within two hours after GVBD (47). Consistent with these results, egg Ca^2+^ oscillations were affected when the channel mediating [Ca^2+^]e influx was abnormal. TRPM7-like channels expressed in oocytes were capable of mediating Ca^2+^ influx and spontaneous Ca^2+^ oscillations disappeared when the channels were blocked by chemical inhibitors (48). The oocyte-specific deletion of *Trpm7* in mouse oocytes resulted in a significant reduction in [Ca^2+^]e influx in GV oocytes (49). These results imply that both spontaneous Ca^2+^ oscillations and maturation of oocytes require the involvement of [Ca^2+^]e. In addition, the prolonged culture in a medium without exogenous Ca^2+^ resulted in chromatin decompression and spindle disintegration in oocytes, which were defined as “interphase-like” (23, 29). All these experimental results demonstrate that extracellular fluid plays a crucial role in oocyte maturation and serves as a significant source of Ca^2+^ during this process.

#### 2.2.3 Mitochondria store

Mitochondria are not only the energy powerhouse of the cell but also can serve as a source of Ca^2+^ in certain cellular processes. Indeed, mitochondria play a pivotal role in cell fate due in large part to their participation in the dynamic regulation of [Ca^2+^]i (50). [Ca^2+^]i homeostasis controlled by mitochondria is also crucial for the appropriate maturation of oocytes and the acquisition of competence (51, 52). During oocyte maturation, active mitochondrial Ca^2+^ ([Ca^2+^]m) was higher around the nucleus than in the mitochondrial cortical layer, suggesting that higher [Ca^2+^]m around chromosomes may play a potential role in stimulating mitochondrial energy to promote calmodulin-responsive oocyte spindle formation (53). Recent evidence suggest that mitochondrial calcium uniporters (MCU) are essential for meiotic progression in mouse oocytes (54). MCU knockdown (KD) by injecting a specific MCU siRNA into fully grown oocytes at the GV stage caused a significant reduction in the proportion of GVBD oocytes and significantly decreased the proportion of MII oocytes. This was consistent with the results of using Ru360, a specific inhibitor of MCU. Further research found that MCU KD resulted in low levels of [Ca^2+^]m, which led to a decline in cytosolic ATP levels, activating and phosphorylating AMP‐activated protein kinase (AMPK). Excessive activation of AMPK resulted in adverse effects on the resumption of meiosis. Together, these findings indicate that the [Ca^2+^]m homeostasis maintained by MCU is necessary for the orderly meiotic maturation of oocytes (54). Similar to this study, Zhang et al. separately targeted *Micu1/Micu2* and the mitochondrial Na^+^/Ca^2+^ exchanger (*NCLX*) for KD with siRNA injected into oocytes to generate the mitochondrial overload model in mouse oocytes. As a consequence, the deficiency of Micu1/Micu2 or NCLX induces increased levels of [Ca^2+^]m in mouse oocytes. Moreover, overload [Ca^2+^]m resulted in a delay in meiosis maturation and mitochondrial dysfunction (55). These results highlight the critical role of [Ca^2+^]m regulation in maintaining mitochondrial function and oocyte maturation. Except for MCU and NCLX, there is another protein named multidrug resistance transporter-1 (MDR-1) expressed in the oocyte mitochondrial membrane. The researchers found that thapsigargin (TG)-induced Ca^2+^ release from MDR-1 mutant MII oocytes did not change significantly, whereas Ca^2+^ ionophore-evoked Ca^2+^ release was significantly increased. These results indicate that MDR-1 mutant oocytes exhibit a greater Ca^2+^ rise, suggesting the accumulation of internal Ca^2+^, most likely in the mitochondria. MDR-1 dysfunction in mouse oocyte quality is attributed to several factors, including [Ca^2+^]m homeostasis. This dysfunction leads to a notable delay in the GV to GVBD transition, chromosome misalignments and a significantly altered meiotic spindle shape (56). Taken together, the Ca^2+^ homeostasis controlled by mitochondria is essential for oocyte maturation and abnormal mitochondrial function can lead to oocyte development disorders.

In conclusion, ER stores, extracellular fluid and mitochondria are the main sources and regulators of Ca^2+^ during oocyte maturation. [Ca^2+^]e or [Ca^2+^]i affects the formation of the first polar body of oocytes, the first meiotic division, and the maintenance of chromosome and spindle conformation between M I and M II in a unique way.

## 3 Calcium homeostasis in GC and CC

Unlike FSH, the luteinizing hormone receptor (LHR) is not expressed in oocytes, but in GC of large follicles. LHRs have been detected in GC of human (57), bovine (58), and pig (59), which were weakly expressed in mice (60) and rats (61). In the GC of intact mouse ovarian follicles, LH can cause persistent Ca^2+^ oscillations that occurred for at least 6 h. This phenomenon is very interesting, but the function of these persistent Ca^2+^ oscillations in oocyte maturation still needs further investigation (20). *In vitro* experiments on sheep and cattle have shown that LH can stimulate an increase of Ca^2+^ in CC, followed by an increase in Ca^2+^ in oocytes (62, 63). The elevation of Ca^2+^ in oocytes starts in the cortex and then spreads throughout the oocyte, suggesting that the increase of Ca^2+^ in the oocyte can be transmitted within the oocyte, but the source of the increase in [Ca^2+^]i in the oocyte remains unknown. The increased Ca^2+^ may be due to the release of [Ca^2+^]i stores (63), or it may be a combination of [Ca^2+^]i stores release and [Ca^2+^]e influx (62). Moreover, in cultured cumulus-oocyte complexes, the activation of the epidermal growth factor (EGF) receptor by LH elevated [Ca^2+^]i in CC, leading to a decrease in natriuretic peptide receptor 2 (NPR2) affinity and cGMP levels. This resulted in the meiotic resumption of mouse oocytes (64). In this study, EGF, another crucial factor for oocyte maturation, was also found to increase [Ca^2+^]i in CC, consistent with previous research (65). In addition, depolarization may also contribute to the increase of Ca^2+^ in CC. LH-induced depolarization was also observed in the CC of sheep (66) and pigs (67), which can activate the L-type Ca^2+^ channel responsible for mediating Ca^2+^ influx into CC, increasing [Ca^2+^]i. Unlike in sheep and bovine, mouse cumulus-oocyte complexes or GC did not show an increase in Ca^2+^ when stimulated by FSH or LH (68). This difference may be due to the different densities of LHR in CC among different species. The long-term effects of LH on Ca^2+^ in mouse CC require further observation, as LH may influence Ca^2+^ in mouse CC gradually and persistently.

Similar to the LHR, the highly conserved ER transmembrane protein named transmembrane and coiled coil domains 1 (TMCO1), which acts as a Ca^2+^ load-activated Ca^2+^ (CLAC) channel (69), is also important for maintaining Ca^2+^ homeostasis in the Ca^2+^ stores of GC (70). *TMCO1* gene mutation can induce autosomal recessive TMCO1 defect syndrome, which belongs to the human cerebrofaciothoracic (CFT) dysplasia spectrum (71, 72). In addition to the main clinical CFT dysplasia spectrum, female mice with *Tmco1* gene KO exhibited progressive loss of follicles, impaired follicle development, and subfertility, resembling the symptoms of premature ovarian failure in females. Based on the function of TMCO1, the researchers found that Ca^2+^ signaling was abnormal in response to ionomycin, TG and ATP, which were about 1.3 to 1.5-fold larger in GC of KO female mice compared to wild type (WT). Interestingly, when TMCO1 was deficient, the ER Ca^2+^ stores in GV and MII oocytes were normal despite the altered pattern of spontaneous Ca^2+^ oscillations. These results suggest that TMCO1 maintenance of Ca^2+^ homeostasis in the ER Ca^2+^ stores of GC is fundamental for follicle development and reproduction, and that disruption of this homeostasis leads to impaired follicle development and reduced fertility (70).

In summary, further research is needed to investigate including, 1) identification and physiological function studies of calcium signaling regulatory molecules, such as calcium channels and calcium pumps in GC and CC; 2) pathological significance of the abnormalities of calcium signaling regulatory molecules in GC and CC; 3) upstream and downstream signaling networks of calcium signaling regulatory molecules involved in cellular function and fate in GC and CC; 4) discovery of new physiological factors regulating calcium homeostasis in GC and CC and reveal pathological and exogenous factors affecting calcium homeostasis in the two types of cells and their corresponding interventions or preventive measures; 5) elucidate the molecular mechanisms by which calcium homeostasis in GC and CC affects oocyte maturation and fertilization; and 6) analyze the similarities and differences, interactions, and their corresponding physiological functions of calcium signaling regulation among the GC, the CC, and the oocyte.

## 4 Ca^2+^ oscillations in fertilization

Oocyte activation (OA) is the process by which oocytes arrested in metaphase II are stimulated to resume meiosis (73). Ca^2+^ signaling is universally recognized as the primary marker of OA in all sexually reproducing species. The sperm-induced pattern of Ca^2+^ oscillations in mammals is relatively stable, with [Ca^2+^]i in the egg rising more than 10-fold shortly after sperm-egg fusion and maintaining this level for several minutes before returning to baseline levels. *In vitro* experiments have shown that Ca^2+^ oscillations occurred in mature oocytes after 20-35 minutes of sperm addition (74). OA occurred with the onset of Ca^2+^ oscillations, and it taken approximately 1-2 hours for all oocytes to be activated. After 4-6 hours of Ca^2+^ oscillations, pronuclei were formed (75). Then, the Ca^2+^ oscillations disappeared and the fertilization was complete. During egg fertilization, each Ca^2+^ oscillation may have its own specific role (75). Additionally, the frequency of Ca^2+^ oscillations is crucial for fertilization (76). These findings suggest that the precise regulation of Ca^2+^ signaling during fertilization is essential for obtaining well-developed embryos and healthy offspring. Therefore, understanding the mechanisms regulating Ca^2+^ oscillations in eggs during fertilization is essential for studying female fertility, as abnormal Ca^2+^ signaling can impact fertility.

The Ca^2+^ oscillations generated in mammalian eggs after fertilization can be artificially divided into two processes, including the increase and maintenance of [Ca^2+^]i in the eggs after sperm-egg fusion and the decrease of [Ca^2+^]i, and restoration of baseline levels. Since Ca^2+^ signaling is regulated by different Ca^2+^ channels and transporters, and the same Ca^2+^ channel or transporter may participate in the regulation of egg activation, fertilization, or embryonic development, we will discuss the role of each Ca^2+^ channel or transporter separately after sperm-egg fusion to avoid repetition.

### 4.1 Calcium elevation and maintenance

#### 4.1.1 Phosphoinositide-specific phospholipase C

**Figure 1:**
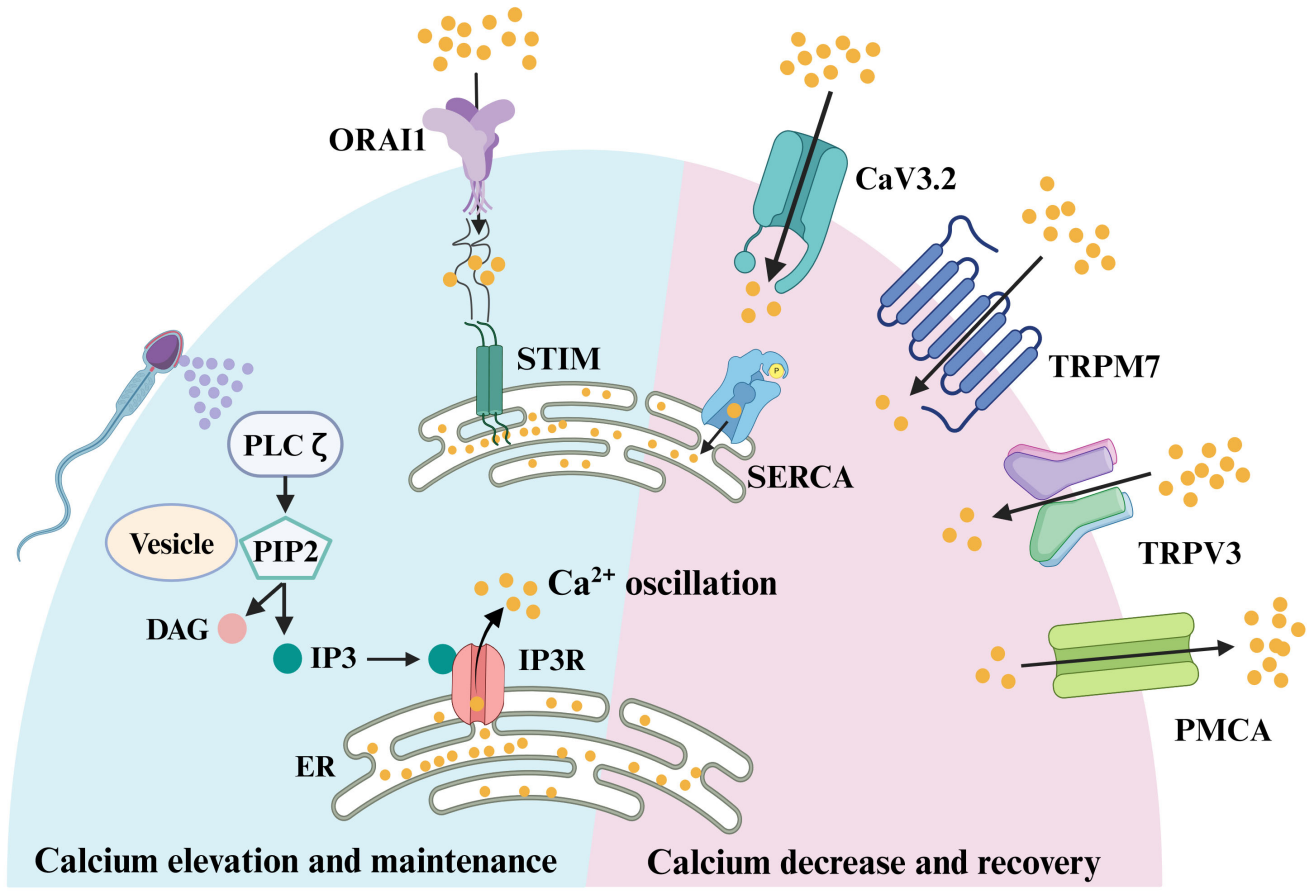
**Signaling pathways involved in oocyte fertilization of mammals.** Sperm factor PLCζ acts on PIP2 in intracellular vesicles to generate IP3, which stimulates IP3R-mediated Ca^2+^ release from ER Ca^2+^ store. Once the ER store is emptied, the egg replenishes the depleted ER store by influx of extracellular Ca^2+^, which was known as store-operated Ca^2+^ entry (SOCE). The main molecular mediators of SOCE are STIM proteins and ORAI proteins. In response to a reduction in ER Ca^2+^, the STIM proteins interact directly with ORAI channels, inducing Ca^2+^ influx. This Ca^2+^ is subsequently pumped back into the ER by the action of sarco-ER Ca^2+^ ATPases (SERCA). Meanwhile, to avoid cytotoxicity from excessive intracellular Ca^2+^ in oocytes, plasma membrane Ca^2+^ ATPase (PMCA) pumps transport some of the Ca^2+^ out of the cytoplasm. When the excess Ca^2+^ in the cytoplasm are excreted, the egg needs to take in some Ca^2+^ to balance the intracellular ion level, and this process is mainly achieved through voltage-gated Ca^2+^ channels, CaV3.2, and transient receptor potential channels, TRPV3 and TRPM7.

In most of the species studied, spermatozoa release sperm factor phospholipase C (PLC) after sperm-egg fusion and subsequently hydrolyze phosphatidylinositol 4,5-bisphosphate (PIP2) by PLC to generate IP3, which is required for Ca^2+^ release during fertilization. The specific PLC varies among species, and there are six families of PLC enzymes in animals, including PLCβ, PLCγ, PLCδ, PLCϵ, PLCζ, and PLCη (77). PLCγ was the first specific PLC enzyme identified to activate and produce IP3 upon fertilization (78) and has been studied in several species (78–81). It was found that tyrosine kinase is the most common mechanism for activating PLCγ (82). However, studies in mice have shown that eggs require multiple sperm factors for activation, but not PLCγ (83). Further analysis showed that PLCζ cloned from a cDNA library of mouse sperm was convincingly identified as the protein in sperm extracts with the capable of activating oocytes (**Figure 1**) (84). Consistent with this result, KD of PLCζ protein in mouse spermatozoa by RNA interference resulted in an abnormal pattern of egg Ca^2+^ oscillations and premature termination during *in vitro* fertilization (IVF). Further analysis of the genotypes of the transgenic mice offspring revealed that no new transgenic mice were born, suggesting that the absence of Ca^2+^ oscillations during fertilization may lead to the failure of proper development of fertilized eggs (85). This finding aligns with previous research indicating that normal Ca^2+^ oscillations support long-term embryo development (86). PLCζ full-length KO mice exhibited normal spermatogenesis and sperm motility. However, KO mouse sperm failed to induce egg Ca^2+^ oscillations either by intracytoplasmic sperm injection (ICSI) or IVF, and an increased rate of polyspermy was observed after IVF or *in vivo* fertilization, with males exhibiting severely reduced fertility (87, 88). Human sperm lacking PLCζ have also been identified in infertile men, with patients exhibiting failure to activate eggs by ICSI, and the same failure to induce Ca^2+^ release by injecting human sperm into mouse eggs (89). In recent years, an increasing number of novel PLCZ1 mutations or abnormalities associated with the failure of human egg activation have been identified (90–92). According to the latest research, a maltose binding protein (MBP)-tagged recombinant human PLCζ protein is capable of inducing Ca^2+^ oscillations in mouse oocytes similar to those observed at fertilization. MBP-PLCζ did not alter embryonic viability compared to the control, providing a first indication of its safety. These results suggest that recombinant MBP-PLCζ protein holds promise for the treatment of OA failure, which can be more precisely controlled compared to the previously discovered strategy of activating oocytes by direct injection of PLCζ RNA, since the stabilization of the RNA upon entry into the cell and the efficiency of protein translation are difficult to control (93). These studies demonstrate the importance of PLCζ in the initiation of Ca^2+^ oscillations and in normal mammalian embryonic development (**Figure 1**).

It was found that there are two sources of PIP2 in eggs, one from the plasma membrane (PM) and the other from intracellular vesicles. There was evidence indicating that PIP2 levels at the PM did not decrease upon fertilization (94, 95). Moreover, fertilization-induced Ca^2+^ oscillations were disrupted in the absence of intracellular vesicle-associated PIP2 (94, 95), suggesting that the PIP2 within intracellular vesicles served as the substrate for PLCζ action (**Figure 1**). However, the question of how PLCζ alters intracellular IP3 ([IP3]i) concentrations during Ca^2+^ oscillations remains unanswered. Whether the underlying [IP3]i level rises with [Ca^2+^]i or if the persistently elevated IP3 level drives the Ca^2+^ oscillations. A recent study addressed this question by detecting a sustained increase in [IP3]i after fertilization of mouse eggs using the FRET method, and it remained high throughout the oscillation. Notably, there was a slight increase in [IP3]i with each increase in [Ca^2+^]i, reflecting the high Ca^2+^ sensitivity of PLCζ (96). However, the peak of [IP3]i occurred after the onset of [Ca^2+^]i elevation. Thus, it was hypothesized that the periodic rise of [Ca^2+^]i in fertilization-induced oscillations was mainly determined by changes in IP3R1 sensitivity (97), which exerted an important role in triggering the [Ca^2+^]i rising phase (96) and influx of Ca^2+^ from the extracellular matrix and thus filling the Ca^2+^ stores (98). In conclusion, PLCζ released from sperm initiates OA and elevates the Ca^2+^ oscillations after sperm-egg fusion.

#### 4.1.2 IP3Rs

Under normal physiological conditions, PLC-triggered egg Ca^2+^ oscillations begin after sperm fuse with eggs (**Figure 1**). During this process, IP3R is the first to respond to PLC (**Figure 1**). Mammalian oocytes, eggs and cells surrounding the eggs express all three isoforms of IP3R (38), but oocytes and eggs predominantly express the IP3R1 isoform (99, 100). The opening of IP3R requires all three subunits to bind to IP3, along with Ca^2+^ (101, 102). Ca^2+^ regulates IP3-induced Ca^2+^ release in a pendulum-like pattern, with IP3-induced Ca^2+^ release being stimulated at low [Ca^2+^]i but inhibited at high [Ca^2+^]i (97). The dual regulation of IP3R (Ca^2+^ and IP3) makes it particularly well-suited to support long-term Ca^2+^ oscillations. In sea urchin eggs, researchers first found increased IP3 levels after fertilization (103), and when IP3 was artificially injected into eggs it could induce Ca^2+^ release from [Ca^2+^]i stores (104), as observed in hamster oocytes (105). When an antibody that functionally blocks the IP3 receptor was injected into hamster oocytes using the microinjection technique, it was found that the antibody completely suppressed sperm-induced Ca^2+^ waves and Ca^2+^ oscillations (106), and that fertilized eggs exhibited polyspermy and late pronucleus formation was inhibited, indicating that IP3-mediated Ca^2+^ release is necessary for both early and late events of mouse egg activation (107). As research progressed, the role of IP3R1 in fertilization in other species was also discovered (108). When mouse fertilized eggs transition from MII to interphase, fertilization-induced Ca^2+^ oscillations become less frequent and cease at the time of pronucleus formation (109). In this process, IP3R1 decreases by about half through ligand-induced hydrolysis (100). Recent study (110) has reported that basal levels of labile Zn^2+^ are essential for sperm-induced Ca^2+^ oscillations in mouse eggs, because Zn^2+^ depleted states evoked by cell-permeable chelators abolished Ca^2+^ responses induced by fertilization and other physiological and pharmacological agonists. It was also found that eggs with lower labile Zn^2+^ content, despite stable store and IP3R1 mass, had decreased IP3R1 sensitivity and decreased ER Ca^2+^ leakage. Zn^2+^ replenishment resumed Ca^2+^ oscillations, but excess Zn^2+^ prevented and aborted these oscillations, thereby compromising IP3R1 responsiveness. These results suggest that oocyte Ca^2+^ responsiveness and IP3R1 function need a window of Zn^2+^ concentration to ensure an optimal response to fertilization and OA (110). Since IP3R1 is embryonic lethal in constitutive KO mice, current studies on this channel have mainly relied on chemically synthesized agonists and inhibitors. As an important ion channel that maintains post-fertilization Ca^2+^ oscillations in eggs, the mechanisms by which IP3R1 regulates oocyte development and post-fertilization Ca^2+^ oscillations remain to be investigated in oocyte conditional knockout (cKO) mice. Comprehensive study of cKO mice is conducive to exploring the significance of IP3R1 abnormalities in female infertility. It is expected to elucidate the role and mechanism of IP3R1, especially downstream molecules/events that receive IP3R regulation in oocyte activation, which will provide new perspectives for understanding the physiology of human oocytes, exploring the causative factors of embryo development failure after normal fertilization, and developing diagnostic and therapeutic tools by detecting or manipulating the activity of IP3R for female infertility.

#### 4.1.3 STIM and ORAI

During egg Ca^2+^ oscillations mediated by sperm factor PLC, the IP3R receptor releases Ca^2+^ from the Ca^2+^ stores. Subsequently, the egg replenishes the depleted ER store through an influx of [Ca^2+^]e, a process known as store-operated Ca^2+^ entry (SOCE) (111, 112). The mechanism of SOCE to replenish ER Ca^2+^ stores as soon as they are empty has been confirmed in somatic cell studies (112, 113). The main molecular mediators of SOCE are STIM proteins and ORAI proteins, with STIM sensing Ca^2+^ levels within the ER Ca^2+^ stores and ORAI proteins acting as Ca^2+^ channels on the PM. There are two STIM proteins, STIM1 and STIM2, and three ORAI proteins, ORAI1, ORAI2, and ORAI3, in mammals. In response to a reduction in ER Ca^2+^, STIM proteins oligomerize and undergo redistribution within the ER to regions closely opposed to the PM, known as ER-PM junctions (**Figure 1**). There, the STIM proteins interact directly with ORAI channels, inducing Ca^2+^ influx (**Figure 1**). This Ca^2+^ is subsequently pumped back into the ER by the action of sarco-ER Ca^2+^ ATPases (SERCA) (**Figure 1**). Whether SOCE is an important physiological mediator in Ca^2+^ influx after fertilization remains contradictory. Earlier studies explored the function of SOCE mainly by employing inhibitors of SOCE and found that SOCE was not required for fertilization in mice (114, 115). To further clarify the physiological function of SOCE, researchers generated STIM1 and STIM2 oocyte cKO mice and obtained STIM1 and STIM2 double KO females by mating. Both fertility and ER Ca^2+^ stores were normal in single or double KO females, with no significant effects on Ca^2+^ influx or Ca^2+^ oscillations during fertilization. These results suggest that in mouse oocytes, neither STIM1 nor STIM2 is an essential ion channel for Ca^2+^ influx when the ER Ca^2+^ store is exhausted (116). Global KO female mice of ORAI1 are also fertile (116, 117), and likewise, there is no significant difference in either ER Ca^2+^ store or Ca^2+^ influx after Ca^2+^ stores are exhausted in MII eggs of Orai1 KO mice. Thus, Orai1 is also not necessary for normal Ca^2+^ signaling in mouse oocytes and eggs. Perhaps due to species differences, the results in pig oocytes are in contrast to studies in mice, where STIM1 and ORAI1 are required for activation of pig oocytes (118). Based on the present findings, it appears that in mice, SOCE is not the mechanism responsible for replenishing ER stores after fertilization and that SOCE may be applicable in other animals. However, we are not sure whether the lack of significant changes in calcium signaling after ORAI1 and STIM KO is due to the compensation by other channels.

There are many other channels involved in the regulation of calcium elevation and maintenance, such as the calcium sensing receptor, ryanodine receptors, and sodium/Ca^2+^ exchangers (13). However, it is yet to be determined whether they are expressed or play significant roles in oocytes.

### 4.2 Calcium decrease and recovery

For Ca^2+^ oscillations to be sustained and to avoid cytotoxicity from excessive [Ca^2+^]i in oocytes, redundant [Ca^2+^]i needs to be removed rapidly to terminate signaling and return Ca^2+^ to baseline levels, and refill the Ca^2+^ stores in anticipation of the next Ca^2+^ transient (**Figure 1**). The main channels responsible for transporting excessive Ca^2+^ out of the cytoplasm in the oocyte membrane is plasma membrane Ca^2+^ ATPase (PMCA) pumps. In addition, the Sarco-ER Ca^2+^ ATPase (SERCA) pumps act as Ca^2+^ pumps on the ER, recycling cytoplasmic Ca^2+^ back into the Ca^2+^ stores against the concentration gradient to prepare for the next Ca^2+^ oscillation. When the excess Ca^2+^ in the cytoplasm is excreted, the egg needs to take in some Ca^2+^ to balance the intracellular ion level, and this process is mainly achieved through voltage-gated Ca^2+^ channels and TRP channels such as TRPV3 and TRPM7. So in the next section, we will discuss how these ion channels reduce excess [Ca^2+^]i and restore basal Ca^2+^ levels in the oocyte.

#### 4.2.1 PMCA

As one of the P-type Ca^2+^ pumps, PMCA maintains [Ca^2+^]i homeostasis by transporting excessive [Ca^2+^]i to the extracellular (**Figure 1**). The mammalian PMCA is encoded by four genes (*Atp2b1-4*) that generate approximately 30 isoforms through variable splicing. PMCA1 and PMCA4 are commonly expressed and are considered to be housekeeping PMCAs (119). PMCA2 is expressed in the nervous system and mammary gland, whereas PMCA3 is expressed in the nervous system (119). In Xenopus oocytes and eggs, the predominant presence of PMCA on the cell membrane of oocytes was demonstrated using antibodies that recognize the four isoforms of mammalian PMCA (120). *Atp2b1-3* can all be detected in mouse GV stage oocytes (121), which is consistent with microarray data (122–124). In mouse oocytes, the expression profiles of *Atp2b1* and *Atp2b3* were almost identical, but as fertilized eggs developed into blastocysts, *Atp2b3* expression became almost undetectable, while *Atp2b1* increased instead (121). To gain more insight into the role of the *Atb2b1* gene in female mouse oocytes, the researchers crossed the *Atb2b1* ^loxP/loxP^ transgenic mice with *Zp3-Cre* mice to obtain female mice with oocyte-specific *Atb2b1* gene disruption before the first meiotic division. Consequently, deletion of the *Atp2b1* isoform did not result in compensatory up-regulation of the alternative isoform and the female fertility was not affected. However, PMCA did have an effect on the Ca^2+^ oscillations in the oocyte during fertilization. The frequency of Ca^2+^ oscillations was not significantly altered in KO mouse oocytes within 1 h of fertilization, but the first Ca^2+^ transients were significantly longer than in wild type (WT) oocytes. Furthermore, oocytes deficient in PMCA1 resulted in larger Ca^2+^ stores (121). This is similar to the results of post-fertilization oocyte Ca^2+^ oscillations studied using the PMCA inhibitor gadolinium (Gd^3+^) (115, 125). Although PMCA1 KO in mouse oocytes did not affect female fertility, abnormal Ca^2+^ at fertilization have long-term effects on offspring growth and the body composition of male mice (121). Follow-up studies are expected on PMCA3 in oocytes and the impact of PMCA1 and PMCA3 on Ca^2+^ oscillations during fertilization.

#### 4.2.2 SERCA

SERCA is also a P-type Ca^2+^ pump located on the ER membrane that transports Ca^2+^ from the cytoplasm to the ER lumen against a concentration gradient (**Figure 1**). In mammals, it is encoded by three genes (*Atp2a1-3*) that generate three major isoforms (SERCA1-3) (126). SERCA2 is the dominant isoform present during fertilization. In mouse oocytes, SERCA2B is the predominantly expressed isoform (125). SERCA can be inhibited by general P-type ATPase inhibitors, such as La^3+^ and orthovanadate, as well as the specific inhibitor TG (126). Oocytes treated with TG showed a significant reduction in the magnitude and duration of the first post-fertilization Ca^2+^ transient and a decrease in the duration of Ca^2+^ oscillations, suggesting an important role for SERCA in post-fertilization Ca^2+^ oscillations (127). Although SERCA2B protein levels remained relatively constant during oocyte maturation, there was a spatial redistribution of the protein, similar to the distribution of ER, from the more diffuse pattern in GV oocytes to the cortical clusters of oocytes (125). Localization of the SERCA pump near the IP3 receptor may facilitate rapid replenishment of ER Ca^2+^ stores after depletion following fertilization. Considering that the current researches are conducted by using SERCA inhibitors, the role of SERCA in oocytes and post-fertilization Ca^2+^ oscillations in mice needed to be further investigated using the cKO models.

#### 4.2.3 Transient receptor potential family and voltage-gated Ca^2+^ channels

Among the PM channels, the mammalian transient receptor potential (TRP) family of channels includes six subfamilies and nearly 30 human members that are expressed in multiple cell types and tissues (128). Two family members of TRP channels have been identified on oocytes and eggs, including TRP vanilloid member 3 (TRPV3) and transient receptor potential cation channel subfamily M member 7 (TRPM7) (**Figure 1**) (129). TRPV3 channels are primarily expressed in the oocyte membrane and their expression increases progressively as the oocyte matures. Activation of TRPV3 by the agonists, carvacrol and 2-APB, induced Ca^2+^ entry, which can parthenogenetically activate eggs (129, 130). TRPV3 channels also mediated Sr^2+^ influx and subsequent egg activation in mice eggs (129), but these results can’t be replicated in human oocytes (131). Although TRPV3 mediates Ca^2+^ entry into eggs, but the Ca^2+^ influx it mediates is not necessary for eggs to maintain Ca^2+^ oscillations, and fertility is not affected in TRPV3 KO females. Then what is the function of TRPV3 in oocytes and eggs? One possibility is that TRPV3 channels constitutively conduct low levels of Ca^2+^ to maintain Ca^2+^ homeostasis. Nevertheless, given the importance of these functions, eggs may have a redundant system that could explain the unchanged oscillation pattern and fertility in TRPV3 KO eggs (129).

TRPM7, expressed in mouse oocytes and eggs (48), is a constitutively active ion channel that is permeable to divalent cations, including Mg^2+^ and Ca^2+^ (132, 133). Nevertheless, the roles of TRPM7-mediated calcium signal have not been investigated and confirmed in human oocytes and eggs. To evaluate the capacity of TRPM7 to mediate Ca^2+^ influx in eggs, the researchers tested the [Ca^2+^]i responses induced by the TRPM7-specific activator Naltriben. MII eggs responded to a higher concentration of Naltriben and the response appears to be sustained. To confirm that [Ca^2+^]i increases induced by Naltriben were caused by Ca^2+^ influx and not due to intracellular off-target effects of the drug, they exposed the cells to Naltriben in media without Ca^2+^. As a consequence, eggs failed to show [Ca^2+^]i increase. These results suggest that Naltriben-induced [Ca^2+^]i increases in eggs were caused by Ca^2+^ influx mediated by TRPM7 channels (48), which was confirmed through the use of TRPM7 cKO female mice. To determine whether TRPM7 mediates Ca^2+^ influx following fertilization, control and *Trpm7* cKO eggs were fertilized while monitoring [Ca^2+^]i. There was no difference in the duration of the first Ca^2+^ transient. However, the oscillation frequency was significantly reduced, which caused abnormal growth in the offspring of *Trpm7* cKO eggs (49). Recent study has also reported that TRPM7-mediated Mg^2+^ influx is indispensable for reaching the blastocyst stage and that TRPM7 underpins Mg^2+^ homeostasis. Additionally, excess Mg^2+^ (but not Zn^2+^ or Ca^2+^) was found to overcome the arrest of *Trpm7*-null embryos (134). However, this study doesn’t evaluate the sperm-induced Ca^2+^ oscillation in eggs. Therefore, we cannot rule out the contributions of TRPM7 in Ca^2+^ influx during Ca^2+^ oscillations. These findings indicate that TRPM7 is a key regulator of Ca^2+^ influx in response to alterations in [Ca^2+^]e and Mg^2+^ ion concentrations and plays an important role in offspring growth.

Voltage-gated Ca^2+^ channels (CaV) intricately regulate the influx of Ca^2+^. CaV can be divided into three major classes based on their structure and function. Among them, the T-type voltage-gated Ca^2+^ channel, CaV3.2, is the ion channel responsible for mediating the typical T-type current in mouse oocytes (**Figure 1**) (135, 136). The *Cacna1h* ^−/−^ females have a reduced litter size. The frequency and amplitude of Ca^2+^ oscillation in *Cacna1h* ^−/−^ eggs were not significantly different compared to those in *Cacna1h* ^+/−^ eggs during IVF. However, the duration of the first Ca^2+^ transient in *Cacna1h* ^−/−^ eggs was significantly shorter than that in *Cacna1h* ^+/−^ eggs. Reduced persistence of Ca^2+^ oscillation in *Cacna1h* ^−/−^ eggs suggests that CaV3.2 channels contribute to Ca^2+^ influx required for sustained Ca^2+^ oscillations at fertilization (137). While in some eggs lacking CaV3.2, Ca^2+^ oscillations can persist long after fertilization. This is likely due to the complementation of other ion channels that mediate Ca^2+^ influx in the oocyte, such as TRPV3 and TRPM7. To further investigate the role of these channels in oocyte development and maturation, the double knockout (dKO) mouse model was generated.

Double-null mice lacking *Trpv3* and *Cacna1h* genes are subfertile (138). Compared to the WT eggs, the time to initiation of oscillations was longer, and the mean number of Ca^2+^ transients was lower in the dKO eggs after fertilization. These data suggest that TRPV3 and CaV3.2 channels are not necessary for the initiation of fertilization, but they remarkably affect the periodicity of such oscillations. Therefore, they are physiological contributors to the [Ca^2+^]i responses during mouse fertilization. They also found that the functional TRPM7 channel is present and upregulated in eggs of dKO mice. TRPM7-like currents are higher in dKO eggs, and Ni^2+^ influx is also enhanced in dKO eggs. Therefore, it is possible that the alteration in the expression and/or function of TRPM7 in dKO eggs is adequate for the initiation and persistence of the low-frequency oscillations triggered by fertilization (138).

Combined loss of TRPM7 and CaV3.2 dramatically alters Ca^2+^ signals and impairs female mice fertility (49). Monitoring of [Ca^2+^]i during IVF revealed that, consistent with the decrease in Ca^2+^ stores, the duration of the first Ca^2+^ transient and the frequency of Ca^2+^ oscillations following IVF were significantly shorter or lower in the dKO eggs compared to eggs lacking only CaV3.2. Interestingly, most of the dKO eggs began oscillating again almost 1 hour after the first Ca^2+^ transient. These findings indicate that CaV3.2 and TRPM7 support the majority of Ca^2+^ influx into eggs during the first hour following fertilization. However, other Ca^2+^ channels should be subsequently activated to allow for the Ca^2+^ oscillation “restart” (49). Whether the other Ca^2+^ channel is TRPV3 is not investigated in this article. These researches indicate that TRPV3, TRPM7 and CaV3.2 play a synergistic role in the process of egg Ca^2+^ oscillation after fertilization. However, the present study showed that deletion of either one (TRPV3/TRPM7/CaV3.2) or both (TRPV3 and CaV3.2 or TRPM7 and CaV3.2) channels did not completely eliminate the egg Ca^2+^ oscillations after fertilization and the corresponding KO females were not completely infertile. These may be due to redundant effects between ion channels. Therefore, further studies are needed to confirm the role of the three channels through double or triple KO mice. With any luck, new ion channels may be discovered during this process.

## 5 Partners and pathways in oocyte Ca^2+^ signaling

**Figure 2:**
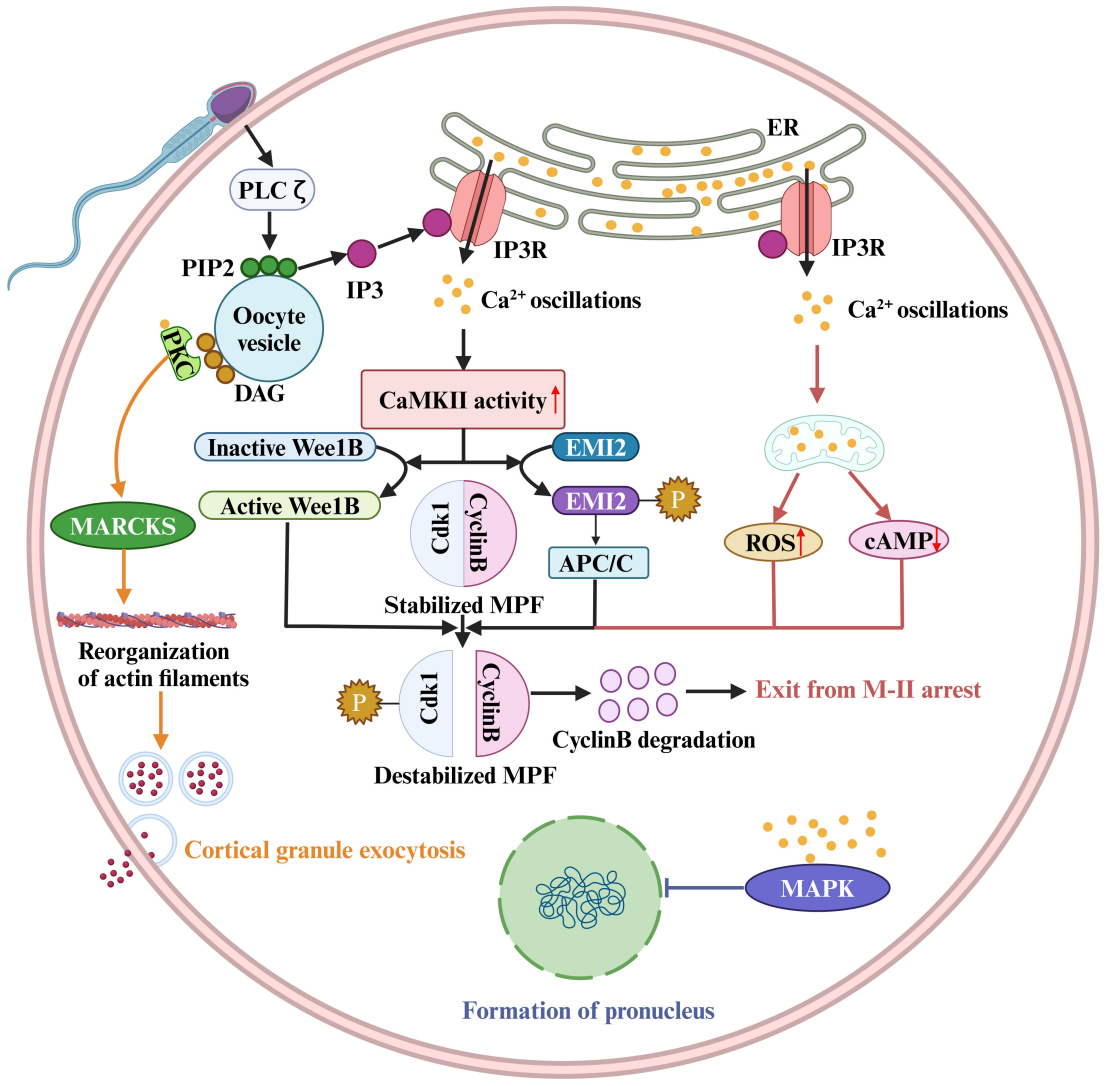
Sperm factor PLCζ acts on PIP2 in intracellular vesicles to generate IP3, which stimulates IP3R-mediated Ca^2+^ release from ER Ca^2+^ store. Ca^2+^ oscillations activate the Ca^2+^/calmodulin-dependent protein kinase II (CaMKII), leading to WEE1B activation. The activate WEE1B phosphorylates CDK1 and inhibits MPF activity. In addition, CaMKII activation results in EMI2 phosphorylation, and phosphorylated EMI2 loses its ability to inhibit the anaphase promoting complex or cyclosome (APC/C). As a result, APC/C disrupts EMI2 and cyclin B, leading to a loss of MPF activity. The destabilized MPF triggers an exit from M-II arrest. Protein kinase C (PKC) binds to synthetic DAGs, which acts on myristoylated alanine-rich C kinase substrate (MARCKS), resulting in reorganization of cortical actin filaments and cortical granule exocytosis. The pronucleus formation of the oocyte also depends on a decrease in MAPK activity. In mouse fertilized eggs, MAPK activity begins to decline when Ca^2+^ oscillations last for 2 hours and gradually declines for several hours after pronuclear formation.

In addition to these channels or Ca^2+^ pumps that directly regulate Ca^2+^ signaling, other regulatory molecules that indirectly affect the Ca^2+^ pump or downstream pathways regulated by Ca^2+^ signaling are also critical for OA and fertilization (**Figure 2**). The best-characterized event of mammalian OA is the resumption and completion of meiosis. However, high levels of M-phase promoting factor (MPF) activity, mainly consisting of cyclin B and a cyclin-dependent kinase (CDK1), maintain meiotic arrest (**Figure 2**) (139). Therefore, a protein or a signal is needed to reduce MPF activity. It has been reported that a moderate increase in [Ca^2+^]i within physiological range induces the generation of reactive oxygen species (ROS), leading to a decrease of cAMP levels (**Figure 2**) (18). Abnormalities in ROS, cAMP, and Ca^2+^ may induce meiotic instability. Instability in the meiotic cell cycle can lead to spontaneous exit from M-II arrest, chromosomal scattering, and incomplete extrusion of the second polar body without forming pronuclei. This phenomenon is known as abortive spontaneous ovum activation (140). Moreover, calmodulin-dependent protein kinase II (CAMKII (CAMK2A)) is also a key protein that decreases the MPF activity in the downstream of Ca^2+^ oscillations (141). Microinjection of constitutively active CAMKII into mouse eggs was shown to induce meiotic resumption (142). Furthermore, although eggs from CAMKIIγ KO or KD mice exhibited a normal pattern of Ca^2+^ oscillations after insemination and undergo cortical granule exocytosis, they failed to resume meiosis (143). CAMKII and meiotic resumption are connected by at least two mechanistic pathways. On the one hand, Ca^2+^ oscillations activate CAMKII, leading to EMI2 phosphorylation (144). Phosphorylated EMI2 cannot inhibit the anaphase promoting complex or cyclosome (APC/C). As a result, APC/C disrupts EMI2 and cyclin B, leading to a loss of MPF activity (**Figure 2**) (145). On the other hand, CaMKII activates WEE1B, which phosphorylates CDK1 and inhibits MPF activity (**Figure 2**) (146). Conversely, when Wee1B is downregulated, oocytes fail to form pronuclei in response to Ca^2+^ signaling. This suggests that Wee1B activation in the oocytes is essential for MPF inactivation and cyclin B disruption (146). These data suggest a two-pronged action of Ca^2+^ signaling on reducing MPF activity (**Figure 2**).

The ability of the oocyte to complete meiosis and enter interphase also depends on a decrease in MAPK activity, mainly ERK1 and ERK2 activity (147). Physiologically, the phosphorylated kinase MEK maintains high ERK1/2 kinase activity. It has been shown that the phosphatase inhibitors or the injection of constitutively activated MEK can prevent the decrease in MAPK (ERK1/2) activity, thereby inhibiting pronuclear formation (147). In contrast, the MEK inhibitor U0126 induces pronuclear formation (148). In mouse fertilized eggs, MAPK activity begins to decline when calcium oscillations last for 2 hours and gradually decreases for several hours after pronuclear formation (149). However, the decline in MAPK kinase activity is not synchronized with MPF inactivation, and experimental results indicate that a decrease in MAPK kinase activity is detected approximately 2 hours after MPF inactivation (150). The delayed decline in MAPK activity may be due to an increase in protein kinase activity (149), but the underlying molecular mechanism is unclear.

Cortical granule exocytosis is a pivotal event in oocyte fertilization. The cortical response can alter the properties of the oocyte PM and zona pellucida to prevent polyspermy. It was found that the increase in [Ca^2+^]i in oocytes induced by electrical pulses triggers the release of most cortical granules (151). However, the signaling of cortical granule exocytosis is distinct from that of meiotic resumption, because injection of constitutively active CAMKII triggers meiotic resumption and pronuclear formation, but not exocytosis (142, 143, 152). In addition, oocytes from *CAMKII^-/-^* mice or WEE1B KD oocytes do not undergo meiotic resumption after fertilization, but in both cases cortical granule exocytosis occurs (143, 146, 153). Thus, meiotic resumption and cortical granule exocytosis appear to be independent downstream events in the early differentiation of the Ca^2+^ signaling pathway.

Numerous studies have shown that mammalian oocyte exocytosis is closely linked to protein kinase C (PKC). Increasing PKC activity, for example, by using synthetic DAGs, can stimulate exocytosis in mammalian eggs (154). The detectable PKC stimulation in the oocyte PM may be primarily due to an increase in Ca^2+^ (155, 156). One idea is that the PKC and calmodulin pathways converge through the translocation of myristoylated alanine-rich C kinase substrate (MARCKS). Exocytosis requires the reorganization of the actin cytoskeleton and the translocation of vesicles to the PM (157). MARCKS is involved in cortical actin reorganization, cytokinesis in other cell types, and cortical granule exocytosis in the egg (158, 159). Vesicle translocation may involve the Ca^2+^-calmodulin-dependent enzyme myosin light chain kinase (MLCK), which targets myosin II in neuroendocrine cells and is responsible for vesicle translocation to the synaptic membrane (157). MLCK inhibitors such as ML7 inhibit cortical granule exocytosis during mouse egg fertilization (160).

In conclusion, CAMKII appears to act as a Ca^2+^-dependent event that triggers meiotic resumption and it will also be interesting to determine the signaling pathway for exocytosis. What’s more, it is essential to demonstrate the relationship between the reduction in MPF and the decrease in MAPK activity, as it is the last in a series of triggers of OA in mammals.

## 6 Ca^2+^ in assisted reproduction

Ca^2+^ oscillations induced by sperm protein PLCζ govern the OA (13) and activate oocyte complex behaviors including meiosis resumption, second polar body extrusion, pronuclear formation, and cortical granule exocytosis. It is clear that the elimination or disruption of Ca^2+^ release patterns during OA underlies many cases of male infertility and abnormal embryogenesis (161). To this extent, the artificial oocyte activation (AOA) technology aims to induce meiotic resumption in the oocyte by artificially elevating [Ca^2+^]i during the process of assisted reproductive technology. AOA contains different means (**Table 1**), including mechanical, electrical, chemical, or some alternative AOA methods, and each means is associated with unique risks and benefits (174). 

**Table 1:** Application of calcium-related stimulators in AOA.

Types	Stimulation	Mechanism	Reference
Electrical	Voltage electric field	Ca^2+^ influx	(162)
Mechanical	Disruption of membrane		(163)
	Microinjection of calcium		(164)
Chemical	A23187	Single Ca^2+^ transient	(165)
	Ionomycin		(12)
	Ethanol		(166)
	Thimerosal	Multiple Ca^2+^ transients	(167)
	Phorbol esters		(168)
	SrCl2		(169)
Alternative AOA promoters	CHX	Parthenogenetic activation	(170)
	TPEN	Meiosis resumption	(171)
	ROS	Protoplast formation	(172)
	WEE2 cRNA	Complementary of pure WEE2 mutations	(173)

### 6.1 Application of Ca^2+^ in AOA

The application of Ca^2+^ in AOA has been well described (11, 12), so this section will offer a brief overview and update of the relevant contents.

The electrical method involves applying a voltage electric field to the oocyte, causing the charged lipid-protein oocyte membrane bilayer to move and form pores. This process eventually allows [Ca^2+^]e to flow into the cytoplasm. The electrical OA has been successfully employed in bovine and human oocytes (162) and the success of the method depends on the size of the pore and the [Ca^2+^]e concentration. However, electrical stimulation not only causes physical damage to oocytes, but also generates excess ROS (186). Therefore, this method of OA needs to be used with caution. However, it is interesting to note that the measurement of electrical resistance in the cell may also be used as a tool to detect oocyte viability and penetration (12). Thus, although electrical OA may not be the ideal clinical treatment, perhaps some modifications could provide a potential diagnosis of OA.

Mechanical activation refers to two main methods, both of which are based on the destruction of the oocyte. One method, which is more invasive, involves physically disrupting the ER membrane and redistributing mitochondria or manually disrupting the oocyte membrane. This is followed by continuous aspiration of the oocyte cytoplasm, which promotes Ca^2+^ influx. This mechanism certainly causes a great deal of physical disruption, but it may increase the intimate contact of sperm with the intracellular membrane, further increasing the chances of successful OA (12, 163, 187). Another approach is to increase Ca^2+^ influx by puncturing the oocyte or injecting trace amounts of Ca^2+^ directly into the oocyte (164). However, both approaches are difficult to standardize, and this method only induces a single Ca^2+^ increase (161).

Chemical methods are the most commonly used methods for OA. Chemical activators trigger a single Ca^2+^ transient or a series of Ca^2+^ oscillations that mimic physiological Ca^2+^ release in the oocyte. Single Ca^2+^ transient activators include Ca^2+^ ionophores (ionomycin and A23187), ethanol, puromycin (a protein synthesis agent) and 6-dimethyaminopurine (6-DMAP, a protein kinase inhibitor). Ionomycin and A23187 are the most commonly used for AOA in IVF cycles (12, 165). The use of ionophores has been shown to increase fertilization and pregnancy rates (188–191). Eggs can also be artificially activated with ethanol. Recent studies have shown that the calcium-sensing receptor (CaSR) is involved in ethanol-induced activation (EIA) (166). EIA has been achieved in mouse (192), bovine (193), and porcine (194) oocytes, however, it does not work in human oocytes (195). In addition, puromycin and 6-DMAP are usually used in combination with other chemical reagents to activate oocytes (196–198).

There are other agents that promote multiple Ca^2+^ transients, including thimerosal (167), phorbol ester (168), or strontium chloride (SrCl_2_) (169). Thimerosal increases the sensitivity of the IP3 receptor to Ca^2+^ primarily by oxidizing sulfhydryl groups, thereby inducing calcium oscillations similar to those induced by sperm (199). Previous studies have shown that thimerosal can activate human oocytes (200–202), resulting in Ca^2+^ oscillations. However, thimerosal is not commonly used because it induces the oxidation of tubulin, which disrupts polymerization and spindle formation (203). Phorbol ester can activate mouse oocytes by inducing sustained oscillations in cell Ca^2+^ (168). However, there have been no relevant applications and no new research progress on phorbol esters in recent years. SrCl_2_ is the preferred compound for OA in rodents (204). Sr^2+^ also induces OA in bovine, porcine, and equine species, but the activation rate and embryo development are low compared to other activation methods (205–208). Despite its more frequent use in animal models, the role of SrCl_2_ in human OA remains controversial (131, 209).

### 6.2 Supplemental measurements of AOA

There are several drugs that target molecules in the Ca^2+^ downstream pathway, known as alternative AOA promoters. Some of these drugs include cycloheximide (CHX) (170), N,N,N’,N’-tetrakis(2-pyridylmethyl) ethane-1,2-diamine (TPEN) (171), roscovitine (172), and WEE2 complementary RNA (WEE2 cRNA) (173). These drugs induce maturation-promoting factor inactivation and meiosis resumption and have been tested for their potential in treating fertilization failure. CHX, a non-specific inhibitor of cell cycle protein B synthesis, has been used to induce parthenogenetic activation in bovine and equine mammalian oocytes (208). In addition, the application of CHX in combination with ionomycin to human oocytes significantly improved the fertilization rate from less than 10% to approximately 50% (170). TPEN is a zinc-specific chelator (210) that induces human oocytes from the MII phase of meiosis to enter the mitotic cycle (171). Roscovitine, a specific inhibitor of cyclin-dependent protein kinases, can cause protoplast formation in oocytes arrested in the MII phase due to Wee1B KD by inducing maturation-promoting factor inactivation (146). However, roscovitine must be combined with ionomycin to successfully activate oocytes (172). *WEE2* cRNA can be applied to human oocytes with pure *WEE2* mutations, which result in fertilization failure, to obtain *in vitro* blastocysts (173), but the effect on late embryonic development has not been reported. However, the above drugs are still in the early stages of clinical trials and their safety requires further discussion.

## 7 Adverse stress-induced Ca^2+^ related ovary dysfunction

Studies have provided evidence that stressors, such as maternal nutrition, environmental contaminants, food-origin toxins and inflammatory disease, have a deleterious effect on the oocyte and ovarian GC. For example, food-origin toxins such as mycotoxins induce ROS production, leading to oxidative damage in oocytes (211). The oocyte achieves its developmental competence through the lengthy process of folliculogenesis. Therefore, exposing animals to these stressors can reduce the ability of oocytes to mature, be fertilized, and develop into viable embryos. Although the intracellular and molecular mechanisms of disruption appear to be multifactorial in nature, oocyte mitochondria are key targets for a variety of stressors.

### 7.1 Ovarian dysfunction due to oocytes damage

Simazine, glyphosate and rotenone are widely used pesticides. Exposure to these substances has been shown to increase [Ca^2+^]i in oocytes, resulting in mitochondrial dysfunction, ROS accumulation and impairment of oocyte maturation (175–177). Consequently, simazine and rotenone induced early apoptosis (175, 177), while glyphosate impaired ER function and disrupted lysosomal function for autophagy (176) (**Table 2**). Phenanthrene (PHE) is one of the most abundant polycyclic aromatic hydrocarbons (PAHs) found in the environment. *In vitro* experiments showed that PHE significantly reduced the percentage of GVBD and the first polar body extrusion, suggesting that PHE impaired meiotic maturation in mouse oocytes. Furthermore, PHE treatment resulted in mitochondrial dysfunction and increased [Ca^2+^]i in oocytes (178) (**Table 2**). Perfluoroalkyl substances (PFHxS), as man-made chemicals with a wide range of consumer and industrial applications, are versatile and resistant to environmental and metabolic degradation. In the *in vitro* experiment, PFHxS decreased the ovulation rate in female mice, but didn’t alter the oocytes morphology. In terms of mechanism, the [Ca^2+^]i decreased in mature oocytes exposed to PFHxS *in vivo*. The researchers hypothesize that this effect is probably due to the deregulation of the ER modulator, Stim1 (179) (**Table 2**). Perfluorodecanoic acid (PFDA) is one of the PFHxS, but it appears to function differently. PFDA impaired porcine oocyte viability and maturation rates in a concentration-dependent manner, which might be associated with the high levels of [Ca^2+^]i. Moreover, gap junction intercellular communication between the CC and the oocyte was disrupted. The effects of PFDA on oocyte Ca^2+^ homeostasis and intercellular communication seem to be responsible for the inhibition of oocyte maturation and oocyte death (180) (**Table 2**). Bisphenol A (BPA), a representative endocrine disrupter mimicking estrogen-action, can affect spontaneous Ca^2+^ oscillations during oocyte maturation (22) and disturb oocyte meiosis (181) (**Table 2**). Some drugs used for treatment can also cause damage to oocytes. Dihydroartemisinin (DHA), an artemisinin derivative, is a highly potent anti-cancer medication. The study found that DHA inhibited porcine oocyte polar body extrusion, blocked cell cycle progression, and impaired early embryo development which were attributed to elevated levels of intracellular and mitochondrial Ca^2+^ (182) (**Table 2**).

**Table 2:** Adverse stress-induced Ca^2+^ related ovary dysfunction.

Cell	Substances	Mechanism	Effects on cells
Oocyte	Simazine	Increased [Ca^2+^]i, mitochondrial dysfunction, ROS accumulation (175)	Decreased oocyte maturation competence and embryonic developmental capacity
	Glyphosate	Increased [Ca^2+^]i, mitochondrial dysfunction, ROS accumulation, impaired ER function and disrupted lysosomal function (176)	Meiotic defects
	Rotenone	Increased [Ca^2+^]i, mitochondrial dysfunction, ROS accumulation (177)	Impaired mouse oocyte maturation and early embryo cleavage
	Phenanthrene	Increased [Ca^2+^]i, mitochondrial dysfunction (178)	Reduced the percentage of GVBD and the first polar body extrusion
	Perfluoroalkyl substances	Decreased [Ca^2+^]i (179)	Decreased the ovulation rate
	Perfluorodecanoic acid	Increased [Ca^2+^]i (180)	Impaired oocyte viability and maturation rates
	Bisphenol A	Affected spontaneous Ca^2+^ oscillations during oocyte maturation (22, 181)	Disturbed the development of granulosa cells and the meiosis progression of oocytes
	Dihydroartemisinin	Elevated levels of intracellular and mitochondrial Ca^2+^ (182)	Inhibited porcine oocyte polar body extrusion, blocked cell cycle progression, and impaired early embryo development
Ovarian granulosa cells	Silica nanoparticles	Activated IP3R1-mediated Ca^2+^ mobilization, leading to apoptosis (183)	Induced follicular atresia and apoptosis
	Dihydroartemisinin	Increased intracellular and mitochondrial Ca^2+^ levels, leading to decreased cell viability and increased apoptosis (184)	Inhibited granulosa cell viability, enhanced the apoptotic rate
	Bisphenol A	Increased ROS production (185)	Reduced the viability

### 7.2 Ovarian dysfunction due to ovarian granulosa cells damage

Silica nanoparticles (SNPs), a major component of ambient particulate matters, can induce follicular atresia via the promotion of ovarian granulosa cells (OGC) apoptosis. A recent study found that ER stress contributed to SNP-induced ovarian toxicity by activating IP3R1-mediated Ca^2+^ mobilization, leading to apoptosis, in which the PERK-ATF4-CHOP-ERO1α pathway played an important role in the dysfunction of OGC (183) (**Table 2**). The interactions between the oocyte and GC are critical for normal follicular development. It is reported that exposure to DHA increased intracellular and mitochondrial Ca^2+^ levels in OGC, leading to decreased cell viability and increased apoptosis in porcine OGC (184) (**Table 2**). BPA and its analogues, such as bisphenol S (BPS), bisphenol F (BPF), and bisphenol AF (BPAF), are widely used to produce a variety of everyday household items. Huang et al. reported that exposure to these bisphenols reduced the viability and increased ROS production in KGN cells (a granulosa-like tumor cell line). These damages may result from changes in [Ca^2+^]i levels in KGN cells induced by bisphenols (185) (**Table 2**).

In conclusion, when changes in external environmental factors lead to an increase in [Ca^2+^]i, oocytes or GC will exhibit a state of stress, impacting oocyte maturation or early embryo development.

## 8 Conclusions and outlook

This article focuses on recent advances in the study of Ca^2+^ signaling in female reproduction, especially in oocyte maturation and functions. Emerging evidence has shown that abnormal Ca^2+^ signaling directly obstructs oocyte maturation, disturbs meiosis and fertilization, and impairs the function of GC and CC, which further leads to fertility impairment. Although knockout of single or multiple Ca^2+^ channel genes in the oocyte does not result in a complete loss of oocyte Ca^2+^ oscillations or complete sterility in female mice due to mutual compensations between the individual channels or calcium-regulated proteins, there is no doubt that Ca^2+^ signaling is critical for female reproduction. When we explore the role of Ca^2+^ in these fields, many questions need to be addressed. First, we are still unaware of the full repertoire of the existence and functions of Ca^2+^ channels or transporters in mammalian oocytes/eggs. Some important calcium channels and proteins are not well characterized in oocytes/eggs. For example, IP3R is particularly important during Ca^2+^ oscillations after fertilization, but substantial evidence is lacking. Functionally blocking the IP3 receptor *in vitro* can completely suppress sperm-induced Ca^2+^ waves and oscillations (106). However, whether IP3Rs are indispensable in physiological situations remains to be investigated in conditional knockout models or mutated human oocytes. Second, the molecular mechanisms of downstream signaling mediated by Ca^2+^ are largely unclear. CAMKII governs Ca^2+^-dependent meiotic resumption, but other key events during fertilization such as cortical granule exocytosis, maternal mRNA recruitment, and maternal-to-zygotic transition are regulated by Ca^2+^ signaling that remains to be further investigated. Third, NLRP14, a maternal effect factor, is essential for maintaining Ca^2+^ oscillations and early embryonic development (212). This reminds us that other unknown maternal factors might manipulate Ca^2+^ oscillations in oocytes. The search for new maternal factors related to Ca^2+^ oscillations will help us to better understand the failure of fertilization due to abnormal Ca^2+^ oscillations caused by females in the clinic. Fourth, it is important to investigate the functional couplings between multiple Ca^2+^-signaling regulatory molecules. Additionally, understanding the specific role of calcium signaling at different stages of oocyte maturation, fertilization, and the transition process (GV-GVBD-MI-MII) is crucial. Fifth, the roles of calcium signaling in gene expression and silencing during oocyte maturation, gene re-activation after fertilization, and other genetic and epigenetic regulations such as DNA methylation, lncRNAs, phase separation and protein post-translational modifications in female reproduction should be uncovered. Last, the safety and efficacy of AOA remain a concern due to a lack of randomized control trials and follow-up risk monitoring studies. Whether there are significant differences between embryos generated by AOA and those generated by natural fertilization, and the risks of AOA-rescued oocytes on the development and growth of the offspring need to be further evaluated. In conclusion, identifying all the Ca^2+^ ion channels in the oocyte and elucidating their regulatory mechanisms will improve our understanding of oocyte maturation and fertilization. This information might be used to more accurately diagnose female infertility and provide alternative strategies to improve the development of embryos generated from assisted reproductive technology procedures.
